# Altered Brain Regional Homogeneity Following Electro-Acupuncture Stimulation at *Sanyinjiao* (SP6) in Women With Premenstrual Syndrome

**DOI:** 10.3389/fnhum.2018.00104

**Published:** 2018-05-31

**Authors:** Yong Pang, Huimei Liu, Gaoxiong Duan, Hai Liao, Yanfei Liu, Zhuo Feng, Jien Tao, Zhuocheng Zou, Guoxiang Du, Rongchao Wan, Peng Liu, Demao Deng

**Affiliations:** ^1^Department of Acupuncture, First Affiliated Hospital, Guangxi University of Chinese Medicine, Nanning, China; ^2^Department of Radiology, First Affiliated Hospital, Guangxi University of Chinese Medicine, Nanning, China; ^3^Life Science Research Center, School of Life Science and Technology, Xidian University, Xi’an, China

**Keywords:** premenstrual syndrome, acupuncture, sanyinjiao, ReHo, fMRI

## Abstract

**Background**: Premenstrual syndrome (PMS) is a menstrual cycle-related disorder which causes physical and mood changes prior to menstruation and is associated with the functional dysregulation of the brain. Acupuncture is an effective alternative therapy for treating PMS, and sanyinjiao (SP6) is one of the most common acupoints used for improving the symptoms of PMS. However, the mechanism behind acupuncture’s efficacy for relieving PMS symptoms remains unclear. The aim of this study was to identify the brain response patterns induced by acupuncture at acupoint SP6 in patients with PMS.

**Materials and Methods**: Twenty-three females with PMS were enrolled in this study. All patients underwent resting-state fMRI data collection before and after 6 min of electroacupuncture stimulation (EAS) at SP6. A regional homogeneity (ReHo) approach was used to compare patients’ brain responses before and after EAS at SP6 using REST software. The present study was registered at http://www.chictr.org.cn, and the Clinical Trial Registration Number is ChiCTR-OPC-15005918.

**Results**: EAS at SP6 elicited decreased ReHo value at the bilateral precuneus, right inferior frontal cortex (IFC) and left middle frontal cortex (MFC). In contrast, increased ReHo value was found at the bilateral thalamus, bilateral insula, left putamen and right primary somatosensory cortex (S1).

**Conclusions**: Our study provides an underlying neuroimaging evidence that the aberrant neural activity of PMS patients could be regulated by acupuncture at SP6.

## Introduction

Premenstrual syndrome (PMS) is a well-known gynecological disorder in fertile women involving a series of psychological, behavioral and physical symptoms, which periodically appear during the luteal phase of the menstrual cycle and relieve soon after the occurrence of menstruation (Yonkers et al., 2008). Approximately 30%–40% of menstruating females suffer from PMS which significantly impacts their daily lives, and 3%–8% of women meet strict DSM-IV diagnosis criteria for premenstrual dysphoric disorder (PMDD), which is a more serious and disabling form of PMS (Ryu and Kim, 2015). Substantial studies have suggested that the etiology of PMS is associated with the functional dysregulation of the central nervous system (CNS) during the luteal phase (Brooks et al., 2002; Amin et al., 2006; Rapkin and Akopians, 2012; Barth et al., 2014; Gao X. et al., 2014). In addition, many neuroimaging studies have also indicated abnormal neural activity in PMS individuals. For example, previous fMRI studies indicated that PMS patients exhibited aberrant neural activity of the default mode network (DMN), which is a region responsible for self-referential activities, such as evaluating characteristics of external and internal cues, planning the future and remembering the past (De Bondt et al., 2015; Liu et al., 2015), suggesting that the abnormal modulation of the DMN might play a crucial role in the pathology of PMS. Consistent with this finding, our most recent study found that PMS patients have abnormal spontaneous neural activity in the DMN and emotion-related brain regions during the luteal phase of the menstrual cycle (Liao et al., 2017). Therefore, a CNS-related intervention modality might be an important therapeutic approach for relieving symptoms of PMS.

As an important therapeutic modality in complementary and alternative medicine (CAM), acupuncture has demonstrated its effectiveness in relieving the physical and psychological symptoms of PMS (Jang et al., 2014). It has been widely and increasingly applied for relieving symptoms of obstetrics and gynecological conditions, including PMS (Kim et al., 2011). According to traditional Chinese medicine (TCM), *sanyinjiao* (SP6), located 3 cun (1 cun = 3.33 cm) directly above the tip of the medial malleoulus on the posterior border of the tibia, has been shown to ameliorate menstrual-related disorders, including primary dysmenorrhea and PMS (Stux and Bruce Pomeranz, 1996; Chae et al., 2007). SP6 is commonly selected as the acupoint of choice for improving the physical and psychological symptoms of PMS in clinical settings (Cho and Kim, 2010). Previous studies have shown that the modulatory effects of acupuncture on patients are mainly mediated via the CNS (Fang et al., 2009). More specifically, acupuncture treatment may work to alleviate symptoms of PMS by modulating abnormal brain responses of the CNS. However, studies attempting to elucidate the modulatory mechanism of acupuncture on PMS are unclear and insufficient. Therefore, more studies on this topic are needed.

fMRI is an important technique to investigate the functions of human brain (Bifone and Gozzi, 2011; Branco et al., 2016). Additionally, fMRI has the ability to monitor acupuncture-related neural response patterns in humans and provides an opportunity for exploring the neural mechanisms of acupuncture. Numerous fMRI studies on acupuncture have found specific activation patterns in the brain elicited by acupuncture (Hui et al., 2000; Wu et al., 2002; Yoo et al., 2004). Regional homogeneity (ReHo), a widely used method, can detect the similarity of synchrony between the time series of a specific voxel with its nearest neighboring voxels and with the report the intensity of regional spontaneous activity in brain (Zang et al., 2004). fMRI combined with ReHo analysis has been widely and successfully used to investigate the mechanisms of a variety of neuropsychiatric diseases (Liu et al., 2006; Wu et al., 2009; Guo et al., 2011). More specifically, our most recent work demonstrated the feasibility of using ReHo to detect the abnormal patterns of spontaneous neural activity in PMS patients (Liao et al., 2017).

In this study we collected fMRI data before and after electro-acupuncture stimulation (EAS) at acupoint SP6 in females with PMS to investigate whether EAS at SP6 could regulate the aberrant brain activity in PMS patients using ReHo analysis. We further hypothesized that the abnormal neural activity in PMS patients could be regulated through EAS at SP6.

## Materials and Methods

### Ethics Statement

This study conforms to the Declaration of Helsinki and was approved by the Medicine Ethics Committee of First Affiliated Hospital, Guangxi University of Chinese Medicine. Every subject was informed of the experimental procedures and asked to sign an informed consent. This study was registered on http://www.chictr.org.cn, the Clinical Trial Registration Number is ChiCTR-OPC-15005918, and the registration number was obtained on 29/01/2015.

### Participants

This study was a follow-up study based on the results of our previous studies, and used the same patient samples as in our previously published manuscript (Liao et al., 2017). Twenty-three female PMS patients were recruited via advertisements posted at the Guangxi University of Chinese Medicine. Each participant was prospectively screened for two consecutive months and asked to complete a daily rating of severity of problems (DRSP) questionnaire in order to quantify the severity of her premenstrual symptoms (Endicott et al., 2006). Diagnostic criteria for PMS were based on the recommendations and guidelines for PMS (Halbreich et al., 2007), while the Diagnostic and Statistical Manual of Mental Disorders-5th Edition (DSM-5; American Psychiatric Association, 2013) was used to exclude participants with PMDD. An experienced gynecologist diagnosed each patient.

The inclusion criteria were as follows: (1) 18–45 years old, right-handed; (2) regular menstrual cycle separated by 24–35 days; (3) premenstrual symptoms occurring 2 weeks before menses in most menstrual cycles; (4) symptoms disappear shortly following the onset of menses; (5) symptoms interfere with daily functioning and/or relationships and/or cause emotional or physical distress or suffering; (6) symptoms periodically appear in the late luteal phase of menstrual cycle and relieve soon after the middle-follicular phase; and (7) symptoms do not worsen due to another physical or mental chronic disorder. The exclusion criteria were: (1) a history of other diseases, including menopausal syndrome, dysmenorrhea, thyroid disease, mastopathy, gynecological inflammation, hysterectomy or bilateral oophorectomy, cancer, or diabetes; (2) a history of psychiatric disorders, such as schizophrenia, schizoaffective disorder, delusional mental disorder, organic mental disorder, psychotic features coordinated or uncoordinated with mood or bipolar disorder; (3) use of benzodiazepines, steroid compound, or other psychotropic drugs; (4) is lactating or pregnant; (5) has any MRI or acupuncture contraindication; and (6) a history of alcohol or drug abuse or is a smoker.

### Experimental Paradigm

**Figure 1:**
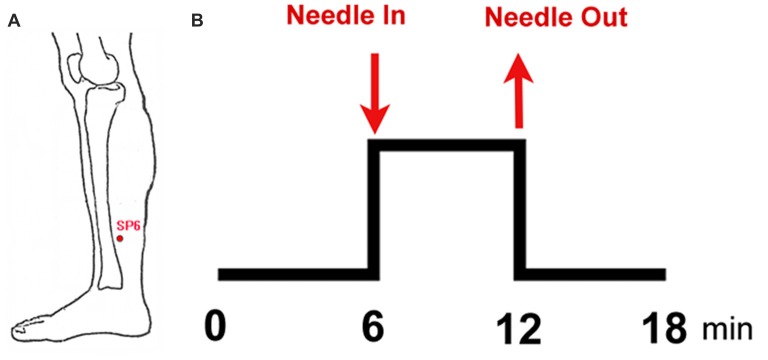
Experimental paradigm. **(A)** Location of sanyinjiao (SP6); **(B)** electroacupuncture stimulation (EAS) paradigm.

This study adopted the non-repeated event-related (NRER) paradigm designed by Qin et al. (2008; Figure 1). Every participant underwent two 6-min fMRI scans, which included a 6-min resting state scan before and after EAS. Acupuncture manipulation was completed by an experienced and licensed acupuncturist (device type: HuaTuo-brand, SDZ-V-type, Shanghai, China). EAS was executed by inserting a stainless-steel disposable needle (specifications: 0.30 mm × 45 mm; Huatuo-brand, Suzhou, Jiangsu, China) into the left leg at acupoint SP6. Another electrode was connected to the acupuncture needle, which was superficially inserted into a point 1.0 cm away from SP6. Due to the physical characteristics of women and sex hormone levels, the test date was arranged during the late luteal phase of the menstrual cycle. All of the tests were performed between 20:00 and 22:00 to ensure a relatively stable and low level of endogenous cortisol and estradiol (Bao et al., 2004). Each subject was informed to “keep their eyes closed, but to stay awake” during the fMRI scan. After the fMRI scan, all subjects were asked to recall Deqi sensations, which are thought to have therapeutic effects in clinical practice, and to complete the visual analog scale (VAS, includes sensations of soreness, numbness, fullness, heaviness, tingling, coolness, warmth, sharp pain, dull pain, aching and pressure; Hui et al., 2005, 2007).

### fMRI Data Collection

fMRI data was collected with a 3.0 tesla MRI system (Magnetom Verio, Siemens Medical, Erlangen, Germany) at the First Affiliated Hospital of Guangxi University of Chinese Medicine. Data was acquired with a single-shot gradient-recalled echo planar imaging (EPI) sequence. The related parameters were as follows: time repetition (TR) = 2000 ms, time echo (TE) = 30 ms, flip angle = 90°, matrix size = 64 × 64, field of view (FOV) = 240 mm × 240 mm, slice thickness = 5 mm and number of slices = 31. High-resolution T1-weighted structural images were collected by a volumetric three-dimensional spoiled gradient recall sequence using the following parameters: TR = 1900 ms, TE = 2.22 ms, flip angle = 9°, matrix size = 250 × 250, FOV = 250 mm × 250 mm, slice thickness = 1 mm and 176 slices.

### Data Preprocessing

The procedures of data preprocessing were identical to our previous work (Liao et al., 2017). Data preprocessing was performed using SPM8 (SPM8)^1^. To ensure the stability of the initial fMRI signal, the first 10 volumes of each time series were removed. Then the remaining functional data was corrected for time delay signals between different slices and realigned to the first volume. Head motion parameters were calculated by assessing the translation in each direction and the angular rotation on every axis for each volume. If the translation/rotation was more than 1.5 mm/1.5°, the data was discarded. The realigned fMRI data was spatially normalized to the montreal neurological institute (MNI) space using the normalization parameters estimated by T1 structural image unified segmentation and was re-sampled to 3 mm × 3 mm × 3 mm voxels. Several sources of spurious variance, such as the estimated motion parameters, average BOLD signals in ventricular and white matter regions, were filtered from the functional images. To abate the effect of low-frequency drifts and high-frequency noise, linear drift was removed and temporal filtering (0.01–0.08 Hz) was applied to the time series of each volume.

### ReHo Analysis

Data preprocessing details can be found in our previous study (Liao et al., 2017). This study used Kendall’s coefficient of concordance (KCC) to measure the synchronization of the time series of a given voxel to its 26 nearest voxels in a voxel-wise way based on the hypothesis that a voxel was temporally similar to the ones of its neighbors. Individual ReHo maps were constructed by calculating the KCC within a gray matter mask in a voxel-wise manner using REST software^2^. The KCC maps were then spatially smoothed via a Gaussian kernel of 6 mm full-width at half-maximum.

### Statistical Analysis

Paired *t*-tests were used to measure patterns of neural activity (ReHo maps) in PMS patients before and after acupuncture at SP6. The contrast threshold was set at *p* < 0.05 (false discovery rate [FDR] corrected) and cluster size >30.

## Results

### Demographic and Clinical Results

Due to obvious head motion, three participants were excluded. Twenty PMS patients were included in the final analysis. The detailed results are shown in Table 1.

**Table 1:** Demographic and clinical characteristics of the study.

Variable	PMS
Number	20
Age (years)	21.85 ± 1.72
BMI	18.60 ± 1.71
DRSP, follicular	39.16 ± 4.15
DRSP, luteal	73.47 ± 7.84
ΔDRSP	34.31 ± 3.69
Menophania (years)	13.75 ± 1.44
Length of menstrual cycle (days)	29.95 ± 1.76
Menstruation (days)	5.60 ± 1.09

*Data are presented as mean ± SD. PMS, premenstrual syndrome; BMI, body mass index; DRSP, daily record of severity of problems; ΔDRSP, difference betweem follicular and luteal phases*.

### Deqi Sensations

**Figure 2:**
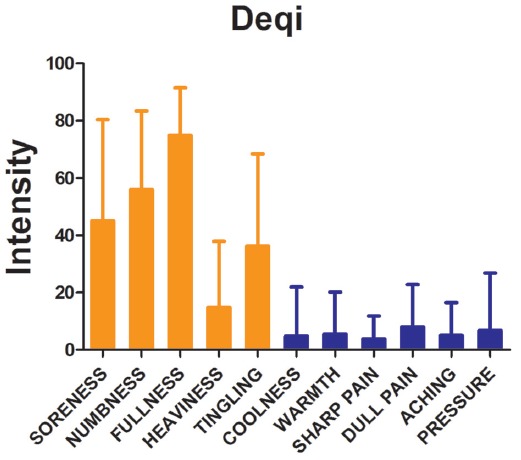
Results of psychophysical analysis. Soreness, numbness, fullness, heaviness and tingling were the most commonly reported Deqi sensations. The error bar stands for standard deviation (SD) of the Deqi sensations.

The main Deqi sensations included soreness, numbness, fullness, heaviness and tingling. Deqi sensations of participants induced by SP6 were expressed as intensity (Figure 2).

### Functional Imaging Results

**Figure 3:**
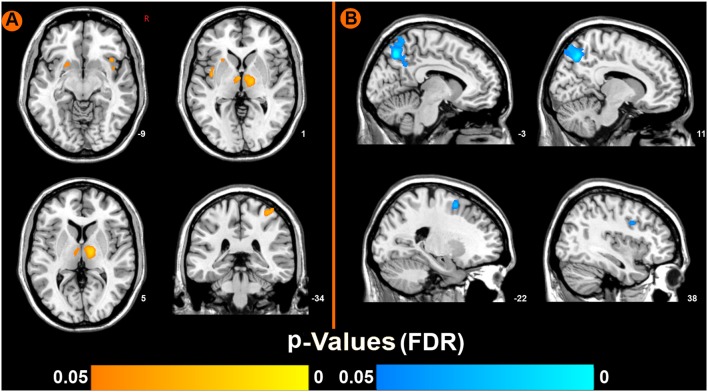
Brain regions showing increased or decreased regional homogeneity (ReHo) values induced by EAS at SP6, compared to pre-acupuncture (*p* < 0.05, false discovery rate corrected). **(A)** Increased ReHo values regions after EAS at SP6. **(B)** Decreased ReHo values regions after EAS at SP6.

Compared to pre-EAS, EAS at SP6 elicited decreased ReHo values at the bilateral precuneus, right inferior frontal cortex (IFC), and left middle frontal cortex (MFC), and increased ReHo values at the bilateral thalamus, bilateral insula, left putamen and right primary somatosensory cortex (S1; Figure 3 and Table 2).

**Table 2:** Main localization of brain maps by comparing electro-acupuncture stimulation (EAS) with resting state in premenstrual syndrome (PMS) patients.

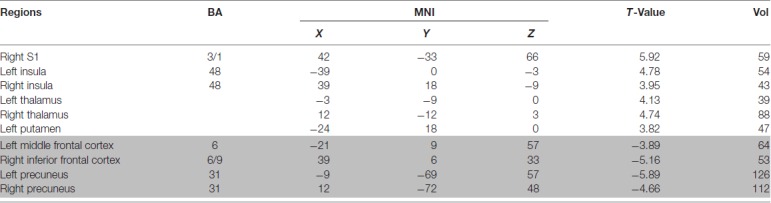

*Abbreviation: BA, Brodmann area; MNI, montreal neurological institute; Vol, voxels; S1, primary somatosensory cortex; PMS, premenstrual syndrome. The highlighted data in gray background are the related brain regions showing decreased ReHo values, and the unhighlighted part are the related brain regions showing increased ReHo values*.

## Discussion

In the present study, we employed fMRI and ReHo analysis to explore EAS induced brain alternations in PMS patients. Our results indicate that EAS at SP6 elicited specific patterns of brain changes during the luteal phase in PMS patients. Our findings provide neuroimaging evidence to better understand the modulatory mechanisms of acupuncture in PMS patients.

As a therapeutic modality in East Asia, acupuncture needles are used to stimulate acupoints on the human body to produce modulation effects. Previous acupuncture neuroimaging studies have shown brain changes in the sensorimotor cortical network (e.g., insula, thalamus, as well as the primary and secondary somatosensory cortex) and the limbic-paralimbic-neocortical network (LPNN; e.g., medial prefrontal cortex, caudate, amygdala, posterior cingulate cortex, precuneus and parahippocampus) induced by acupuncture stimulation (Dhond et al., 2007; Fang et al., 2009; Chae et al., 2013). Our results are consistent with these previous findings. In the present study we elaborated upon this research and investigated changes in neural activity (decreased and increased) using ReHo analysis to illuminate the modulatory effects of acupuncture at SP6 in PMS patients.

### Decreased ReHo Value in the DMN

We found decreased ReHo values in some areas of the DMN, a network that is involved in self-referential activities, such as remembering the past and planning for the future (Raichle and Snyder, 2007; Buckner et al., 2008). The precuneus, a key node of the DMN, is significantly involved in a distributed network with cortical and sub-cortical regions to integrate both self-generated and external information (Cavanna and Trimble, 2006), and is associated with emotion processing (Tanaka and Kirino, 2016). The precuneus appears to induce significant neural changes in response to incongruent stimuli information rather than to congruent stimuli (Kitada et al., 2014). The CNS of PMS patients usually faces incongruent stimuli due to fluctuations in sex hormones (Halbreich et al., 2003). Thus, we conjecture that the decreased ReHo in the precuneus is related to sex hormone fluctuations. In addition, the IFC and MFC, major components of the prefrontal cortex (PFC), are mainly associated with the integration of emotional and cognitive functions (Gusnard et al., 2001; Simpson et al., 2001). In a previous study, investigators found that the function of the PFC in emotional response inhibition was susceptible to changes in the female menstrual cycle (Amin et al., 2006), and that the PFC was more likely to have abnormal neural activity in response to negative emotional stimuli. Our previous study found increased ReHo values in the bilateral precuneus, right IFC, and left MFC, which we refer to as the DMN (Liao et al., 2017), and these results are consistent with other studies (De Bondt et al., 2015; Liu et al., 2015). We speculate that the negative psychological symptoms (e.g., mood swings, anger, impatience and depression) or incongruent stimuli from fluctuations in the menstrual cycle in PMS are due to abnormal DMN activity. Therefore, insights into the modulation of the DMN by invention methods are necessary to understand the therapeutic modulatory mechanisms for PMS. Fortunately, previous studies have suggested that ReHo can be used to measure spontaneous neural activity during a task and thus can measure task activations (Yuan et al., 2013). Moreover, many western medicine studies have suggested that ReHo analysis can provide critical information to better understand CNS-related functional neural synchrony alterations induced by some treatment methods, such as hemodialysis and equine-assisted activities and therapy, and the decreased ReHo values in the DMN have been suggested to be significantly associated with medication modulatory effects on patient’s brain (Chen et al., 2015; Yoo et al., 2016). It is worth noting that in the current study the majority of the decreased ReHo values elicited by acupuncture at SP6 were located in the DMN, including the right IFC, left MFC and bilateral precuneus. These findings indicate that the abnormal neural activity of the DMN was modulated by SP6 in PMS patients and might have helped reestablish normal function to the DMN and thus have rehabilitation implications. The modulation effects of acupuncture and its clinical efficacy also suggests that acupuncture serves a role in maintaining the body’s homeostatic balance (Mayer, 2000). Thus, we speculate that the underlying mechanism of acupuncture on PMS might be attributed to acupuncture intervention effects on re-establishing the normal neural activity of the DMN.

### Increased ReHo Value in Sensorimotor Cortical Network

This study also found increased ReHo values in some brain regions of the sensorimotor cortical network, such as the bilateral thalamus, bilateral insula, left putamen and right S1. The thalamus plays an important role in controlling the flow of information to the cortex (Sherman and Guillery, 2002). It relays motor, sensory, and spatial information to the cortex (Sherman, 2007) and mediates the interaction of attention and arousal (Portas et al., 1998). The spinothalamic tract projects to the thalamus, from which information is transmitted to the S1 and insula, respectively (Anand et al., 2007). The insula receives afferent information from the thalamus, and forms anatomical interconnections with extensive cortical and sub-cortical structures related to higher-order brain functions, including pain perception, memory, and decision-making (Craig et al., 2000; Craig, 2011; Nieuwenhuys, 2012; Zhuo, 2016). The putamen is an important component of the striato–thalamo–cortical circuitry and is related to habitual behavior, action initiation and motivational processing (Graybiel, 2008). Prior studies indicate that pain stimuli are not only in core areas of the afferent neuraxis, including the thalamus, S1 and insula (Coghill et al., 2001; Brooks et al., 2002; Bingel et al., 2003), but also in the motor output system (e.g., putamen), which is responsible for producing spatially guided defensive behavior (Bingel et al., 2002). Moreover, fMRI studies on acupuncture have revealed that brain changes in the sensorimotor cortical network are induced by acupuncture (e.g., insula, thalamus, putamen, as well as S1 (Fang et al., 2009). Davis et al. (1998) demonstrated that the changes in the somatosensory cortex together with the thalamus occurred following somatosensory stimulation at various acupoints. Furthermore, fMRI studies revealed that acupuncture at SP6 could induce changes in neural activity in the sensorimotor cortical network of a sleep-deprived brain (Gao L. et al., 2014). Interestingly, our study also revealed that there was a trend between Deqi sensations and ReHo changes in certain brain regions using a correlation analysis (see the Supplementary Table S1). Several Deqi sensations (Coolness, Warmth, Pressure and Sharp pain) were positively correlated with ReHo changes at the bilateral precuneus, bilateral insula and left putamen. Meanwhile, tingling and numbness were negatively correlated with ReHo changes at the precuneus and insula. To our knowledge, Deqi sensations are complex subjective experiences associated with the sensorimotor cortex. Based on these findings, we speculate that acupuncture at SP6 might have modulatory effects on the sensorimotor cortical network. According to our current results of increased ReHo at the bilateral insula, bilateral thalamus, left putamen, and right S1 following acupuncture at SP6 in PMS patients, we speculate that the widely increased synchronization of neuronal activity in the sensorimotor cortical network may be due to altered sensory transduction pathways in the brain induced by SP6.

There are several limitations in this study. First, the present study only demonstrated increased/decreased ReHo values in the brain of PMS patients modulated by acupuncture at SP6 but did not show that these ReHo values were “PMS specific,” as there were no healthy controls or sham conditions. In the future, the use of a control condition would be advised. Second, although our study indicated that there was a trend between Deqi sensations and ReHo changes of certain brain regions, we were unable to extract any meaningful results from these complicated correlations. Finally, our sample size of PMS patients was not very big; therefore, the present findings should be retested with a larger sample size in the future.

In conclusion, we elaborated on our previous study and used ReHo analysis to investigate the specific fMRI brain response patterns to acupuncture at SP6 in PMS patients during the late luteal phase. Our findings indicate that the abnormal neural activity of the DMN and sensorimotor cortical network in PMS patients could be modulated by acupuncture at SP6. These results provide neuroimaging evidence to better understand the underlying mechanisms of acupuncture at SP6 in PMS patients.

## Author Contributions

DD and YP proposed the theory and designed the experiment, made substantial contributions to the present study, revised and handled the manuscript. HLiu, GDuan and HLiao mainly were mainly responsible for recruiting and evaluating PMS patients. ZF, JT and ZZ mainly for recruiting the PMS patients and treating with acupuncture. GDu and RW performed the MR scan protocols and acquired and stored MRI data. YL and PL conducted the data processing and analysis, interpreted the conceptions of data processing. All of the authors consented the final version to be published.

## Conflict of Interest Statement

The authors declare that the research was conducted in the absence of any commercial or financial relationships that could be construed as a potential conflict of interest.

## Footnotes

^1^http://www.fil.ion.ucl.ac.uk/spm/

^2^http://restfmri.net/forum/index.php

## Supplementary Material

The Supplementary Material for this article can be found online at: https://www.frontiersin.org/articles/10.3389/fnhum. 2018.00104/full#supplementary-material

## References

[B52] American Psychiatric Association (2013). Diagnostic and Statistical Manual of Mental Disorders: DSM-5™. 5th Edn. Arlington, VA: American Psychiatric Publishing, Inc.

[B1] Amin Z. Epperson C. N. Constable R. T. Canli T. (2006). Effects of estrogen variation on neural correlates of emotional response inhibition. Neuroimage 32, 457–464. 10.1016/j.neuroimage.2006.03.013 16644236

[B2] Anand P. Aziz Q. Willert R. van Oudenhove L. (2007). Peripheral and central mechanisms of visceral sensitization in man. Neurogastroenterol. Motil. 19, 29–46. 10.1111/j.1365-2982.2006.00873.x 17280584

[B3] Bao A. M. Ji Y. F. Van Someren E. J. W. Hofman M. A. Liu R. Y. Zhou J. N. (2004). Diurnal rhythms of free estradiol and cortisol during the normal menstrual cycle in women with major depression. Horm. Behav. 45, 93–102. 10.1016/j.yhbeh.2003.09.004 15019795

[B4] Barth A. M. I. Ferando I. Mody I. (2014). Ovarian cycle-linked plasticity of δ-GABAA receptor subunits in hippocampal interneurons affects γ oscillations *in vivo*. Front. Cell. Neurosci. 8:222. 10.3389/fncel.2014.00222 25157218 PMC4128222

[B5] Bifone A. Gozzi A. (2011). Functional and Pharmacological MRI in Understanding Brain Function at a Systems Level. Berlin Heidelberg: Springer. 10.1007/7854_2010_103 21225416

[B6] Bingel U. Quante M. Knab R. Bromm B. Weiller C. Buchel C. (2002). Subcortical structures involved in pain processing: evidence from single-trial fMRI. Pain 99, 313–321. 10.1016/s0304-3959(02)00157-4 12237210

[B7] Bingel U. Quante M. Knab R. Bromm B. Weiller C. Büchel C. (2003). Single trial fMRI reveals significant contralateral bias in responses to laser pain within thalamus and somatosensory cortices. Neuroimage 18, 740–748. 10.1016/s1053-8119(02)00033-2 12667851

[B8] Branco P. Seixas D. Deprez S. Kovacs S. Peeters R. Castro S. L.. (2016). Resting-state functional magnetic resonance imaging for language preoperative planning. Front. Hum. Neurosci. 10:11. 10.3389/fnhum.2016.00011 26869899 PMC4740781

[B9] Brooks J. C. W. Nurmikko T. J. Bimson W. E. Singh K. D. Roberts N. (2002). fMRI of thermal pain: effects of stimulus laterality and attention. Neuroimage 15, 293–301. 10.1006/nimg.2001.0974 11798266

[B10] Buckner R. L. Andrews-Hanna J. R. Schacter D. L. (2008). The brain’s default network: anatomy, function, and relevance to disease. Ann. N Y Acad. Sci. 1124, 1–38. 10.1196/annals.1440.011 18400922

[B11] Cavanna A. E. Trimble M. R. (2006). The precuneus: a review of its functional anatomy and behavioural correlates. Brain 129, 564–583. 10.1093/brain/awl004 16399806

[B12] Chae Y. Chang D. S. Lee S. H. Jung W. M. Lee I. S. Jackson S.. (2013). Inserting needles into the body: a meta-analysis of brain activity associated with acupuncture needle stimulation. J. Pain 14, 215–222. 10.1016/j.jpain.2012.11.011 23395475

[B13] Chae Y. Kim H. Y. Lee H. J. Park H. J. Hahm D. H. An K.. (2007). The alteration of pain sensitivity at disease-specific acupuncture points in premenstrual syndrome. J. Physiol. Sci. 57, 115–119. 10.2170/physiolsci.rp012706 17378970

[B14] Chen H. J. Qi R. Kong X. Wen J. Liang X. Zhang Z.. (2015). The impact of hemodialysis on cognitive dysfunction in patients with end-stage renal disease: a resting-state functional MRI study. Metab. Brain Dis. 30, 1247–1256. 10.1007/s11011-015-9702-0 26146033

[B15] Cho S. H. Kim J. (2010). Efficacy of acupuncture in management of premenstrual syndrome: a systematic review. Complement. Ther. Med. 18, 104–111. 10.1016/j.ctim.2009.12.001 20430293

[B16] Coghill R. C. Gilron I. Iadarola M. J. (2001). Hemispheric lateralization of somatosensory processing. J. Neurophysiol. 85, 2602–2612. 10.1152/jn.2001.85.6.2602 11387404

[B17] Craig A. D. (2011). Significance of the insula for the evolution of human awareness of feelings from the body. Ann. N Y Acad. Sci. 1225, 72–82. 10.1111/j.1749-6632.2011.05990.x 21534994

[B18] Craig A. D. Chen K. Bandy D. Reiman E. M. (2000). Thermosensory activation of insular cortex. Nat. Neurosci. 3, 184–190. 10.1038/72131 10649575

[B19] Davis K. D. Kwan C. L. Crawley A. P. Mikulis D. J. (1998). Functional MRI study of thalamic and cortical activations evoked by cutaneous heat, cold and tactile stimuli. J. Neurophysiol. 80, 1533–1546. 10.1152/jn.1998.80.3.1533 9744957

[B20] De Bondt T. De Belder F. Vanhevel F. Jacquemyn Y. Parizel P. M. (2015). Prefrontal GABA concentration changes in women-Influence of menstrual cycle phase, hormonal contraceptive use, and correlation with premenstrual symptoms. Brain Res. 1597, 129–138. 10.1016/j.brainres.2014.11.051 25481417

[B21] Dhond R. P. Kettner N. Napadow V. (2007). Neuroimaging acupuncture effects in the human brain. J. Altern. Complement. Med. 13, 603–616. 10.1089/acm.2007.7040 17718643

[B22] Endicott J. Nee J. Harrison W. (2006). Daily Record of Severity of Problems (DRSP): reliability and validity. Arch. Womens Ment. Health 9, 41–49. 10.1007/s00737-005-0103-y 16172836

[B23] Fang J. Jin Z. Wang Y. Li K. Kong J. Nixon E. E.. (2009). The salient characteristics of the central effects of acupuncture needling: limbic-paralimbic-neocortical network modulation. Hum. Brain Mapp. 30, 1196–1206. 10.1002/hbm.20583 18571795 PMC6871074

[B25] Gao X. Sun P. Qiao M. Wei S. Xue L. Zhang H. (2014). Shu-Yu capsule, a Traditional Chinese Medicine formulation, attenuates premenstrual syndrome depression induced by chronic stress constraint. Mol. Med. Rep. 10, 2942–2948. 10.3892/mmr.2014.2599 25270424

[B24] Gao L. Zhang M. Gong H. Bai L. Dai X. J. Min Y.. (2014). Differential activation patterns of FMRI in sleep-deprived brain: restoring effects of acupuncture. Evid. Based Complement. Alternat. Med. 2014:465760. 10.1155/2014/465760 25024729 PMC4082872

[B26] Graybiel A. M. (2008). Habits, rituals, and the evaluative brain. Annu. Rev. Neurosci. 31, 359–387. 10.1146/annurev.neuro.29.051605.112851 18558860

[B27] Guo W. B. Liu F. Xue Z. M. Yu Y. Ma C. Q. Tan C. L.. (2011). Abnormal neural activities in first-episode, treatment-naive, short-illness-duration, and treatment-response patients with major depressive disorder: a resting-state fMRI study. J. Affect. Disord. 135, 326–331. 10.1016/j.jad.2011.06.048 21782246

[B28] Gusnard D. A. Akbudak E. Shulman G. L. Raichle M. E. (2001). Medial prefrontal cortex and self-referential mental activity: relation to a default mode of brain function. Proc. Natl. Acad. Sci. U S A 98, 4259–4264. 10.1073/pnas.071043098 11259662 PMC31213

[B29] Halbreich U. Backstrom T. Eriksson E. O’Brien S. Calil H. Ceskova E.. (2007). Clinical diagnostic criteria for premenstrual syndrome and guidelines for their quantification for research studies. Gynecol. Endocrinol. 23, 123–130. 10.1080/09513590601167969 17454164

[B30] Halbreich U. Borenstein J. Pearlstein T. Kahn L. S. (2003). The prevalence, impairment, impact, and burden of premenstrual dysphoric disorder (PMS/PMDD). Psychoneuroendocrinology 28, 1–23. 10.1016/s0306-4530(03)00098-2 12892987

[B31] Hui K. K. Liu J. Makris N. Gollub R. L. Chen A. J. Moore C. I.. (2000). Acupuncture modulates the limbic system and subcortical gray structures of the human brain: evidence from fMRI studies in normal subjects. Hum. Brain Mapp. 9, 13–25. 10.1002/(sici)1097-0193(2000)9:1<13::aid-hbm2>3.0.co;2-f 10643726 PMC6871878

[B32] Hui K. K. Liu J. Marina O. Napadow V. Haselgrove C. Kwong K. K.. (2005). The integrated response of the human cerebro-cerebellar and limbic systems to acupuncture stimulation at ST 36 as evidenced by fMRI. Neuroimage 27, 479–496. 10.1016/j.neuroimage.2005.04.037 16046146

[B33] Hui K. K. Nixon E. E. Vangel M. G. Liu J. Marina O. Napadow V.. (2007). Characterization of the “deqi” response in acupuncture. BMC Complement. Altern. Med. 7:33. 10.1186/1472-6882-7-33 17973984 PMC2200650

[B34] Jang S. H. Kim D. I. Choi M. S. (2014). Effects and treatment methods of acupuncture and herbal medicine for premenstrual syndrome/premenstrual dysphoric disorder: systematic review. BMC Complement. Altern. Med. 14:11. 10.1186/1472-6882-14-11 24410911 PMC3898234

[B35] Kim S. Y. Park H. J. Lee H. Lee H. (2011). Acupuncture for premenstrual syndrome: a systematic review and meta-analysis of randomised controlled trials. BJOG 118, 899–915. 10.1111/j.1471-0528.2011.02994.x 21609380

[B36] Kitada R. Sasaki A. T. Okamoto Y. Kochiyama T. Sadato N. (2014). Role of the precuneus in the detection of incongruency between tactile and visual texture information: a functional MRI study. Neuropsychologia 64, 252–262. 10.1016/j.neuropsychologia.2014.09.028 25281887

[B37] Liao H. Pang Y. Liu P. Liu H. Duan G. Liu Y.. (2017). Abnormal spontaneous brain activity in women with premenstrual syndrome revealed by regional homogeneity. Front. Hum. Neurosci. 11:62. 10.3389/fnhum.2017.00062 28243196 PMC5303726

[B38] Liu H. Liu Z. Liang M. Hao Y. Tan L. Kuang F.. (2006). Decreased regional homogeneity in schizophrenia: a resting state fMRI study. Neuroreport 17, 19–22. 10.1097/01.wnr.0000195666.22714.35 16361943

[B39] Liu Q. Li R. Zhou R. Li J. Gu Q. (2015). Abnormal resting-state connectivity at functional MRI in women with premenstrual syndrome. PLoS One 10:e0136029. 10.1371/journal.pone.0136029 26325510 PMC4556707

[B40] Mayer D. J. (2000). Acupuncture: an evidence-based review of the clinical literature. Annu. Rev. Med. 51, 49–63. 10.1146/annurev.med.51.1.49 10774452

[B41] Nieuwenhuys R. (2012). The insular cortex: a review. Prog. Brain Res. 195, 123–163. 10.1016/B978-0-444-53860-4.00007-6 22230626

[B42] Portas C. M. Rees G. Howseman A. M. Josephs O. Turner R. Frith C. D. (1998). A specific role for the thalamus in mediating the interaction of attention and arousal in humans. J. Neurosci. 18, 8979–8989. 10.1523/JNEUROSCI.18-21-08979.1998 9787003 PMC6793555

[B43] Qin W. Tian J. Bai L. Pan X. Yang L. Chen P.. (2008). FMRI connectivity analysis of acupuncture effects on an amygdala-associated brain network. Mol. Pain 4:55. 10.1186/1744-8069-4-55 19014532 PMC2596101

[B44] Raichle M. E. Snyder A. Z. (2007). A default mode of brain function: a brief history of an evolving idea. Neuroimage 37, 1083–1090; discussion 1097–1089. 10.1016/j.neuroimage.2007.02.041 17719799

[B45] Rapkin A. J. Akopians A. L. (2012). Pathophysiology of premenstrual syndrome and premenstrual dysphoric disorder. Menopause Int. 18, 52–59. 10.1258/mi.2012.012014 22611222

[B46] Ryu A. Kim T. H. (2015). Premenstrual syndrome: a mini review. Maturitas 82, 436–440. 10.1016/j.maturitas.2015.08.010 26351143

[B47] Sherman S. M. (2007). The thalamus is more than just a relay. Curr. Opin. Neurobiol. 17, 417–422. 10.1016/j.conb.2007.07.003 17707635 PMC2753250

[B48] Sherman S. M. Guillery R. W. (2002). The role of the thalamus in the flow of information to the cortex. Philos. Trans. R. Soc. Lond. B Biol. Sci. 357, 1695–1708. 10.1098/rstb.2002.1161 12626004 PMC1693087

[B50] Simpson J. R. Jr. Snyder A. Z. Gusnard D. A. Raichle M. E. (2001). Emotion-induced changes in human medial prefrontal cortex: I. During cognitive task performance. Proc. Natl. Acad. Sci. U S A 98, 683–687. 10.1073/pnas.98.2.683 11209065 PMC14648

[B51] Stux M. G. Bruce Pomeranz P. D. (1996). Basics of Acupuncture. Berlin: Springer Berlin.

[B49] Tanaka S. Kirino E. (2016). Functional connectivity of the precuneus in female university students with long-term musical training. Front. Hum. Neurosci. 10:328. 10.3389/fnhum.2016.00328 27445765 PMC4925677

[B53] Wu M. T. Sheen J. M. Chuang K. H. Yang P. Chin S. L. Tsai C. Y.. (2002). Neuronal specificity of acupuncture response: a fMRI study with electroacupuncture. Neuroimage 16, 1028–1037. 10.1006/nimg.2002.1145 12202090

[B54] Wu T. Long X. Zang Y. Wang L. Hallett M. Li K.. (2009). Regional homogeneity changes in patients with Parkinson’s disease. Hum. Brain Mapp. 30, 1502–1510. 10.1002/hbm.20622 18649351 PMC6871162

[B55] Yonkers K. A. O’Brien P. M. Eriksson E. (2008). Premenstrual syndrome. Lancet 371, 1200–1210. 10.1016/S0140-6736(08)60527-9 18395582 PMC3118460

[B56] Yoo J. H. Oh Y. Jang B. Song J. Kim J. Kim S.. (2016). The effects of equine-assisted activities and therapy on resting-state brain function in attention-deficit/hyperactivity disorder: a pilot study. Clin. Psychopharmacol. Neurosci. 14, 357–364. 10.9758/cpn.2016.14.4.357 27776388 PMC5083948

[B57] Yoo S. S. Teh E. K. Blinder R. A. Jolesz F. A. (2004). Modulation of cerebellar activities by acupuncture stimulation: evidence from fMRI study. Neuroimage 22, 932–940. 10.1016/j.neuroimage.2004.02.017 15193624

[B58] Yuan R. Di X. Kim E. H. Barik S. Rypma B. Biswal B. B. (2013). Regional homogeneity of resting-state fMRI contributes to both neurovascular and task activation variations. Magn. Reson. Imaging 31, 1492–1500. 10.1016/j.mri.2013.07.005 23969197 PMC3873744

[B59] Zang Y. Jiang T. Lu Y. He Y. Tian L. (2004). Regional homogeneity approach to fMRI data analysis. Neuroimage 22, 394–400. 10.1016/j.neuroimage.2003.12.030 15110032

[B60] Zhuo M. (2016). Contribution of synaptic plasticity in the insular cortex to chronic pain. Neuroscience 338, 220–229. 10.1016/j.neuroscience.2016.08.014 27530697

