# Supplementary material for: Altered Brain Regional Homogeneity Following Electro-Acupuncture Stimulation at Sanyinjiao (SP6) in Women With Premenstrual Syndrome
Source: Front Hum Neurosci. 2018 May 31;12:104. doi: 10.3389/fnhum.2018.00104 (PMC5990869; doi:10.3389/fnhum.2018.00104)
Supplement: Supplementary file 1 [file Table_1.DOC]

**Altered Brain** **Regional Homogeneity Following Electro-Acupuncture Stimulation at** ***Sanyinjiao* (SP6) in** **Women with** **Premenstrual Syndrome**

Yong Pang^1^*^†^*, Huimei Liu^1^*^†^*, Gaoxiong Duan^2^*^†^*, Hai Liao^2^*^†^*, Yanfei Liu^2^, Zhuo Feng^1^, Jien Tao^1^, Zhuocheng Zou^1^, Guoxiang Du^2^, Rongchao Wan^2^, Peng Liu^3^, and Demao Deng^2^*

^1^Department of Acupuncture, First Affiliated Hospital, Guangxi University of Chinese Medicine, Nanning, Guangxi, China

| **Table Correlational Analysis Between Deqi Sensations and ReHo change** | | | |
| --- | --- | --- | --- |
| **Brain Regions** | **Deqi Sensations** | **Pearson correlation coefficient** | ***P* value** |
| L_Precuneus | Coolness | 0.536 | 0.015 |
| R_Precuneus | Tingling | -0.451 | 0.046 |
| L_Insula | Warmth | 0.516 | 0.020 |
| R_Insula | Numbeness | -0.455 | 0.044 |
| R_Insula | Pressure | 0.492 | 0.028 |
| L_Putamen | Sharp pain | 0.456 | 0.043 |

^2^Department of Radiology, First Affiliated Hospital, Guangxi University of Chinese Medicine, Nanning, Guangxi, China

^3^Life Science Research Center, School of Life Science and Technology, Xidian University, Xi’an, Shaanxi, China

**Supplement Materials**
